# Suppression of Quackery

**Published:** 1843-05-27

**Authors:** 


### SUPPRESSION OF QUACKERY.

The French Chamber of Peers has passed a law ordaining that, in future, no patents shall be granted for secret remedies or for pharmaceutical preparations.

